# Information Extraction of High Resolution Remote Sensing Images Based on the Calculation of Optimal Segmentation Parameters

**DOI:** 10.1371/journal.pone.0158585

**Published:** 2016-06-30

**Authors:** Hongchun Zhu, Lijie Cai, Haiying Liu, Wei Huang

**Affiliations:** 1 Shandong University of Science and Technology, Geomatics College, Qianwan port road 579, Qingdao, Shandong 266590, China; 2 Shandong University of Science and Technology, The Key Laboratory of Geomatics and Digital Technology, Qianwan port road 579, Qingdao, Shandong 266590, China; 3 Shandong University of Science and Technology, College of Information Science and engineering, Qianwan port road 579, Qingdao, Shandong 266590, China; University California Los Angeles, UNITED STATES

## Abstract

Multi-scale image segmentation and the selection of optimal segmentation parameters are the key processes in the object-oriented information extraction of high-resolution remote sensing images. The accuracy of remote sensing special subject information depends on this extraction. On the basis of WorldView-2 high-resolution data, the optimal segmentation parameters methodof object-oriented image segmentation and high-resolution image information extraction, the following processes were conducted in this study. Firstly, the best combination of the bands and weights was determined for the information extraction of high-resolution remote sensing image. An improved weighted mean-variance method was proposed andused to calculatethe optimal segmentation scale. Thereafter, the best shape factor parameter and compact factor parameters were computed with the use of the control variables and the combination of the heterogeneity and homogeneity indexes. Different types of image segmentation parameters were obtained according to the surface features. The high-resolution remote sensing images were multi-scale segmented with the optimal segmentation parameters. Ahierarchical network structure was established by setting the information extraction rules to achieve object-oriented information extraction. This study presents an effective and practical method that can explain expert input judgment by reproducible quantitative measurements. Furthermore the results of this procedure may be incorporated into a classification scheme.

## Introduction

Modern remote sensing technology is developing rapidly. It presents characteristics that involve multiple platforms, multi-angles, and multi-type sensors, as well as high geometric, temporal, and spectral resolutions. High-resolution remote sensing images are widely used, and the information extraction from high-resolution remote sensing image is an important research direction. High-resolution remote sensing images have high geometric positioning accuracy, good stereo mapping ability, and good flexibility; theyalso provide effective data support for the detailed extraction of spatial objects[1–2]. An automatic, rapid, accurate, and efficient extraction method of classification information from high-resolution remote sensing images is urgently needed for high-resolution remote sensing applications[3].

Many scholars have applied the object-oriented method to extract information from high-resolution remote sensing images because of its rich geometry and the texture characteristics. A series of experimental studies has shown that the object-oriented method can add value to information extraction conducted on the same data using different methods[4–9]. In the object-oriented information extraction from high-resolution images, the segmentation is one of the most important steps. The appropriate segmentation parameters, such as the optimal segmentation scale and the shape and structure factors, are the key factors in image segmentation. At present, the calculation method of the optimal scale mainly uses expert experience, calculation models, objective functions, and so on. For example, Yan[10] presented an object-oriented typical ground object extraction on the basis of multi-level rules and improved the image segmentation method based on region growing. Hu[11]proposed an optimal segmentation-scale calculation model to improve the accuracy of object-oriented image interpretation. Huang[12] developed mean variance and object max-area method to calculate the scale factor. Tian[13] proposed a framework to identify optimal segmentation scale for a given feature type. Yu[14] proposed a new method of optimal segmentation scale selection for the object-oriented remote sensing image classification-vector distance index method. According to the analysis of the above studies, the current calculation methods of segmentation parameter, such as expert experience and object function method[12, 15], focus on the scale factor, depend on expert experience, and is restricted by the lack of mathematical law. Furthermore, the calculation method of the shape and firmness factor is lacking and mainly relies on subjective judgments. Therefore, examining object-oriented high-resolution remote sensing image segmentation, as well as the calculation method of the optimal segmentation parameters and thematic information extraction according to these segmentation parameters, has important the oretical research significance and practical application value. So, the purpose of this study is to present a calculation method of the optimal segmentation parameters and extract classification information from high-resolution remote sensing imagebased on the calculation of optimal segmentation parameters.

This paper presents a new calculation approach for the optimal segmentation parameters of high-resolution remote sensing image, by which global detection and local optimization, quantitative analysis and fuzzy rules are employed to explore image characters. Thereafter, the calculation method of the image segmentation scale, shape factor, and tightness factor is studied. On the basis of experimental data, the optimal parameters of ground objects are obtained. At last, to confirm the effect and accuracy of this proposed new method, the segmentation classification result that uses optimal segmentation parameters is compared with the per-pixel supervised classification result.

## Calculation of the Segmentation Parameters

High-resolution remote sensing imageshave subtle information on surface features, such as reflectance characteristics across a spectrum, texture (descriptive statistics concerning the relationships of pixel values to those of their near neighbours within a circumscribed area), the geometry of features defined by groups of contiguous pixels with similar attributes, and the relationships of these features to others. The identification and classification of different features require the combination of many types of feature information. The classification information extraction of object-oriented images is based on the prior definition of appropriate object-oriented image segmentation. The fractal network evolution algorithm (FNEA) was first proposed by Baatz,M. &A.Schäpe[16], and this method has been adopted by remote sensing software such as eCognition, ENVI and et.al[17]. FNEA integrates the spectrum and spatial heterogeneities of different segmentation objects, and the algorithm achieves the largest homogeneity within object segmentation and the largest average heterogeneity between objects[16]. The heterogeneity value of the image object spectral information is calculated with the spectrum weight, shape information weight, spectral value heterogeneity, and shape heterogeneity. In the actual operations of image segmentation, the parameters focus on band combination weights and the segmentation scale, shape and firmness factors[17].

### Experimental data and platform

**Fig 1:**
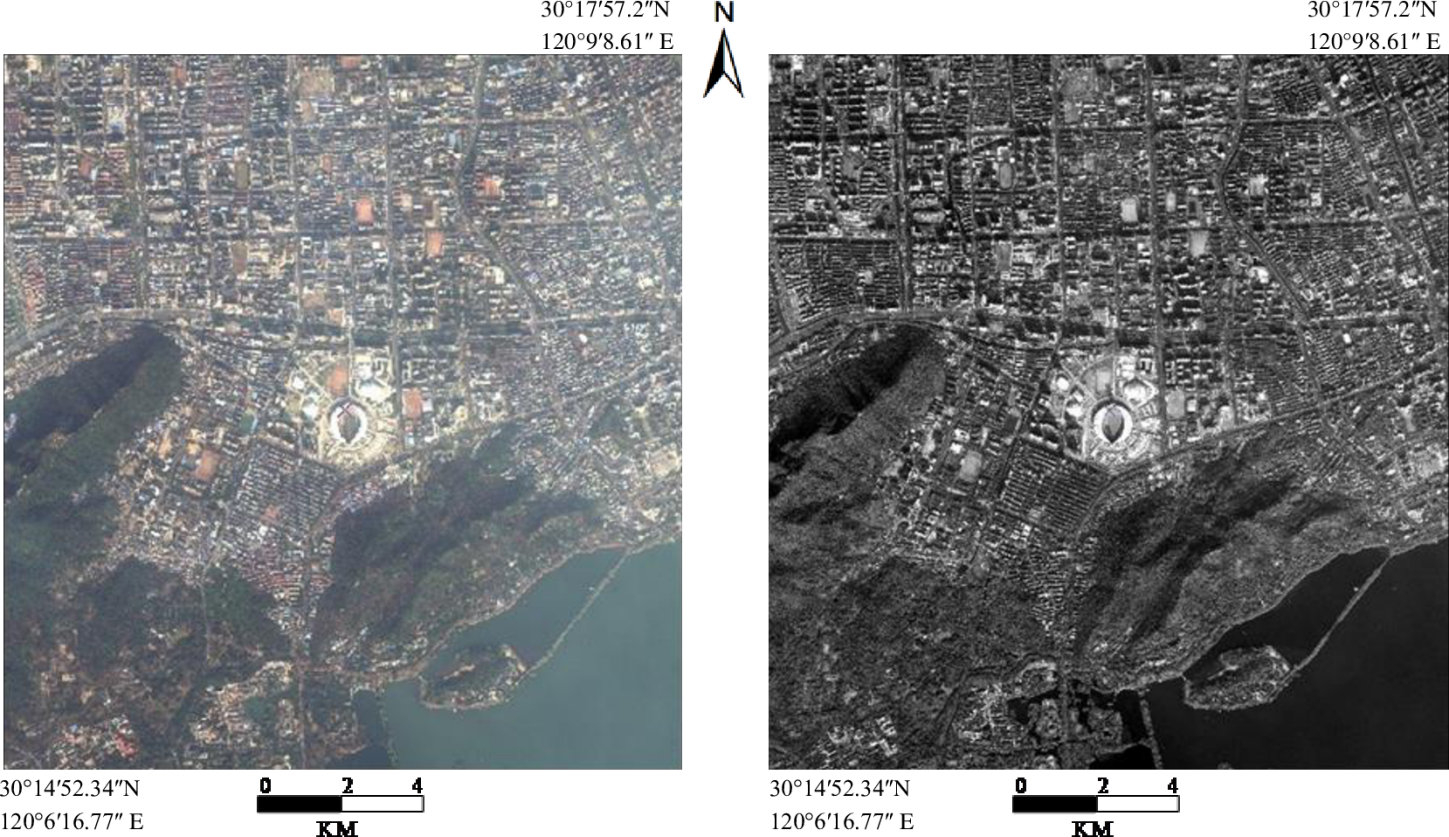
The experimental data. The two sketch maps on the left indicate Natural coloring multi-spectral image of the study area; the right side is panchromatic image of the study area.

The experimental data in this study is WorldView-2 data including multispectral image and panchromatic image, which were obtained on January13, 2010. The spatial resolution of multispectral imageand panchromatic image data are1.8 and 0.5 meter respectively. The study area is located in the Xihu District Hangzhou, China at30°14′52.34″N, 120°6′16.77″ E and 30°17′57.20″N, 120°9′8.61″E, covering an area of approximately 312.43 km^2^ (Fig 1).

The software packages used in this study are MATLAB, ENVI, and eCognition. MATLAB is used in the attributive data processing of the image objects. The assessment standard for the best segmentation parameter is obtained by the suggested calculation model, and the curve graphs of the relevant scaling parameters are drawn. ENVI is mainly used for image pre-processing and evaluation, image cropping, and classification information extraction based on image pixels. eCognition is used to obtain the segmentation attribute properties of ground objects, and the multi-scale segmentation and information extraction of the high-resolution remote sensing images are realized with the optimal segmentation parameters.

### Image enhancement processing

**Fig 2:**
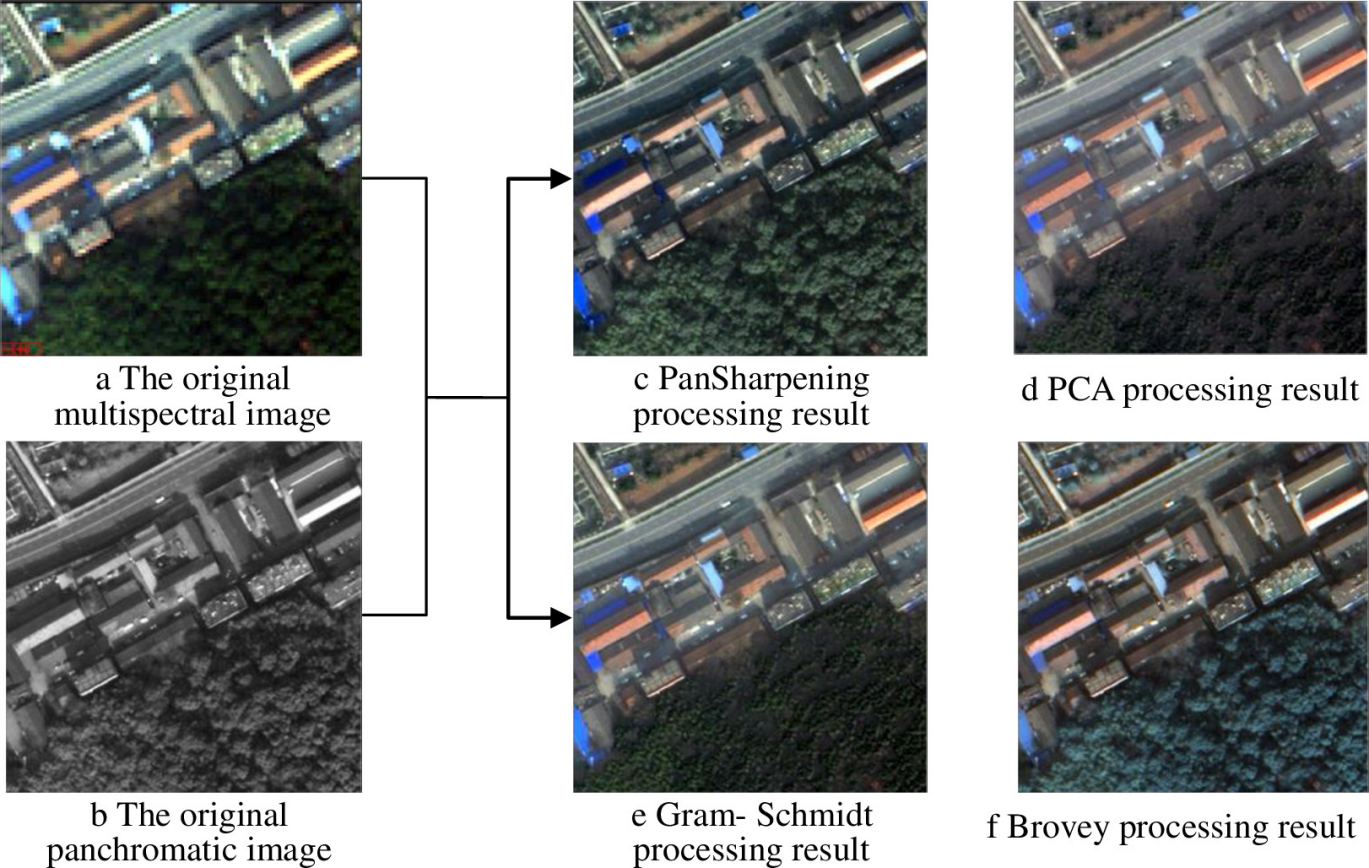
Image enhancement processing results.

Processing requires that all data have a common pixel size. It is desirable to retain spectral values as close as possible to the original, and to benefit maximally from higher spatial resolution panchromatic data. To accomplish this, four commonly-used methods, such as principal component analysis (PCA), Gram–Schmidt transformation, Pan-sharpening fusion, and Brovey transform, were selected and compared. The principle of the four methods is described in the references [18]. The processing results are shown in Fig 2.

Through the qualitative analysis of the four enhancement processes in Fig 2, we find that the object contour, such as roads and buildings, are significantly enhanced. The color of the pan sharpening fusion results has the best effect. The Pan-sharpening and Brovey fusion processing results show good image sharpness and texture. In the PCA and Brovey fusion results, the resolution of roads, buildings, trees, and bare land is considerably improved, and the texture details are enhanced. However, restricted by the processing band, all types of enhancement results demonstrate different degrees of distortion. The texture and detail information of the Pan-sharpening results are better than those of the other results. Furthermore, some quantitative indexes are calculated to objectively evaluate the enhancement results. The findings are shown in Table 1. The four quantitative indexes of different images, which are listed in the first column of Table 1, are the average values of all bands.

**Table 1:** Quantitative index of enhancement results.

	mean value	standard deviation	information entropy	Deviation index
original panchromatic image	391.13	59.00	1.95	
original multispectral image	332.27	50.46	1.95	
PCA processing result	332.29	49.21	1.82	0.10
Gram-Schmidt processing result	332.28	49.82	1.84	0.11
Brovey processing result	129.88	20.05	1.59	0.29
Pan-Sharpening processing result	333.21	53.44	1.86	0.06

Table 1 depicts that the mean value, standard deviation, and information entropy of the Pan-sharpening results are the largest, and the deviation index is minimal. This finding suggests that abundant information and good spectral fidelity effect are found in Pan-sharpening result. Therefore, on the basis of the qualitative and quantitative analysis results, the Pan-sharpening method is used for data pre-processing in this study.

**Fig 3:**
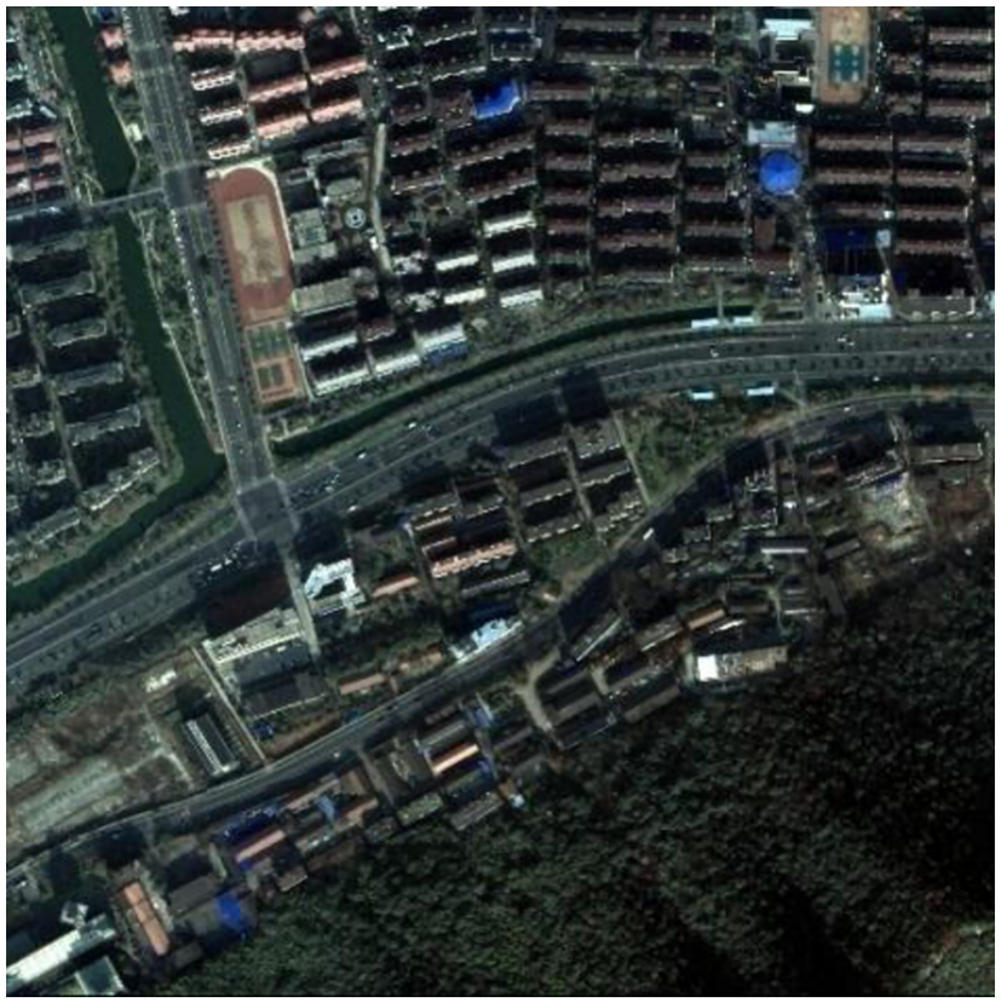
The study area after subset.

To improve the efficiency of the experiment, and given the large amount of data generated after image enhancement processing, this study uses the cut 400*400 pixel image as the experiment data. The cutting result is shown in Fig 3. The cutting experiment data, including the typical urban setting, can be used to calculate the segmentation parameters and perform image classification.

### Calculation of the optimal segmentation scale factor

#### Methodology

At present, the variance method is mainly used to calculate the optimal image segmentation scale[11], but this method only considers polygon object spectral information and does not consider the image’s spectral structure of the object. To address the deficiency of the variance method, this study proposes a weighted variance method of the optimal segmentation scale calculation based on the spectral structure. The applied process is described as follows:

- Calculation of several parameters

The mean value of the single Pan-sharpened band is the average brightness value of all pixels that are affiliated with the same object. The mean variance of the single Pan-sharpened band refers to the variance of the mean value of all the objects. The mean value of object b is presented in Formula (1), and that of the entire image object is presented in Formula (2):

$$C_{Lb}=\frac{1}{n}\cdot \Sigma_{i=1}^{n}C_{Lbi}$$ (1)

$${\bar{C}}_{Lb}=\frac{1}{m}\cdot \Sigma_{i=1}^{m}C_{Lb}$$ (2)

In Formulas (1) and (2), C_Lbi_ is the brightness value of the pixel i of the object b in the band L, n is the number of pixels of one object, C_Lb_ is the brightness average value of object b in the band L, and m is the number of objects.

- Calculation of the weighted variance

Formulas (1) and (2) are used to calculate the average variance of each band. Formula (3) is then used to calculate the weighted variance:

$$S^{2}=\Sigma_{L=1}^{N}t_{L}*S_{L}^{2}$$ (3)

In Formula (3), N is the number of bands, t_L_ is the weight of the band L, and ${\text{S}}_{\text{L}}^{2}$ is the variance of the band L.

- Determination of the segmentation band and its weight

This study uses the analytical method of the optimum index factor (OIF, Formula 4), which was developed by Chavez to determine the optimal band combination[19]. In the OIF method, the optimal band combination is determined according to the standard deviation of the bands and the inter-band correlation coefficient. Generally, the informative bands with small correlations and large spectral differences are selected:

$$\text{OIF}=\frac{\sum_{i=1}^{3}s_{i}}{\sum_{i=1}^{3}\sum_{j=i+1}^{3}\left|R_{ij}\right|}$$ (4)

In Formula (4), *s*_*i*_ is the standard deviation of band i, and *R*_*ij*_ is the correlation coefficient between two arbitrary bands. Calculating the covariance matrix and the correlation coefficient matrix of every two bands obtains the OIF values of different band combinations. The optimal band combination of image segmentation corresponds to the OIF maximum value. At the same time, the segmentation weights of the optimal band combination are determined according to the covariance matrix of every two bands.

- Drawing the corresponding curve scale between the variance and segmentation scale to determine the optimal segmentation scale value

Drawing the corresponding curve scale between the variance and segmentation scale, as well asaccessing the peaks of the curve, determines the optimal segmentation scale value. Combined with the visual interpretation method, the segmentation scale value corresponding to each featuretype is analyzed and obtained.

#### Experiment and results analysis

1. Band combination and its weight

The covariance matrix and correlated coefficient matrix of every two bands of the experiment data were calculated. The results are shown in Tables 2 and 3. The optimal band combination and OIF value of the experiment data are calculated with Formula (5). The results are listed in Table 4.

**Table 2:** Band covariance matrix of study area.

	Band 1	Band 2	Band 3	Band 4	Band 5	Band 6	Band 7	Band 8
Band 1	133.13							
Band 2	343.83	916.42						
Band 3	636.98	1709.7	3352.9					
Band 4	499.95	1330.7	2641.3	2177.6				
Band 5	531.13	1429.7	2851.7	2354.8	2598.1			
Band 6	556.44	1515.6	3193.3	2627.5	2859.7	4049.9		
Band 7	634.01	1781.9	3839.5	3044.3	3331.2	5573.2	8744.7	
Band 8	486.08	1368.4	2937.4	2338.6	2550.8	4312.3	6772.3	5335.2

**Table 3:** Band correlation matrix of study area.

	Band 1	Band 2	Band 3	Band 4	Band 5	Band 6	Band 7	Band 8
Band 1	1.00							
Band 2	0.98	1.00						
Band 3	0.95	0.98	1.00					
Band 4	0.93	0.94	0.98	1.00				
Band 5	0.90	0.93	0.97	0.99	1.00			
Band 6	0.76	0.79	0.87	0.88	0.88	1.00		
Band 7	0.59	0.63	0.71	0.70	0.70	0.94	1.00	
Band 8	0.58	0.62	0.69	0.69	0.69	0.93	0.99	1.00

**Table 4:** The OIF of study area image.

Band combination	OIF	Band combination	OIF	Band combination	OIF
3,5,7	3386.626	3,5,8	3126.68	5,6,8	2934.911
3,7,8	3350.293	4,6,7	3116.223	4,5,8	2909.966
3,6,7	3309.136	3,6,8	3056.632	2,4,7	2887.827
3,4,7	3298.084	3,4,8	3035.3	4,6,8	2859.116
5,7,8	3257.31	2,3,7	3032.239	3,5,6	2808.586
5,6,7	3188.997	2,7,8	2997.406	6,7,8	2805.054
4,7,8	3183.097	2,5,7	2984.53	2,3,8	2757.239
4,5,7	3174.843	2,6,7	2976.376	1,3,7	2749.029

Table 4 shows that the OIF values of bands 3, 5, and 7 are the largest. This result indicates that the combination of bands 3, 5, and 7 that correspond to the largest OIF values have the largest amount of information, and the correlations between bands are minimal. Therefore, the best combination of bands for this experiment is bands3, 5, and 7. The segmentation weight of each band is cited according to the spectral information of each band. Table 2 shows that the variances of bands 7, 3, and 9 are 8744.7, 3352.9, and 2598.1, respectively. The variance of band 7 is approximately three times as much as that of bands 3 and 5. As a result, the weighted values of bands 3 and 5 are set to 1, the weighted value of band 7 is set to 3, and the weighted values of the rest of the bandsare set to 0in the image segmentation experiment.

2. Segmentation scale calculation experiment

**Fig 4:**
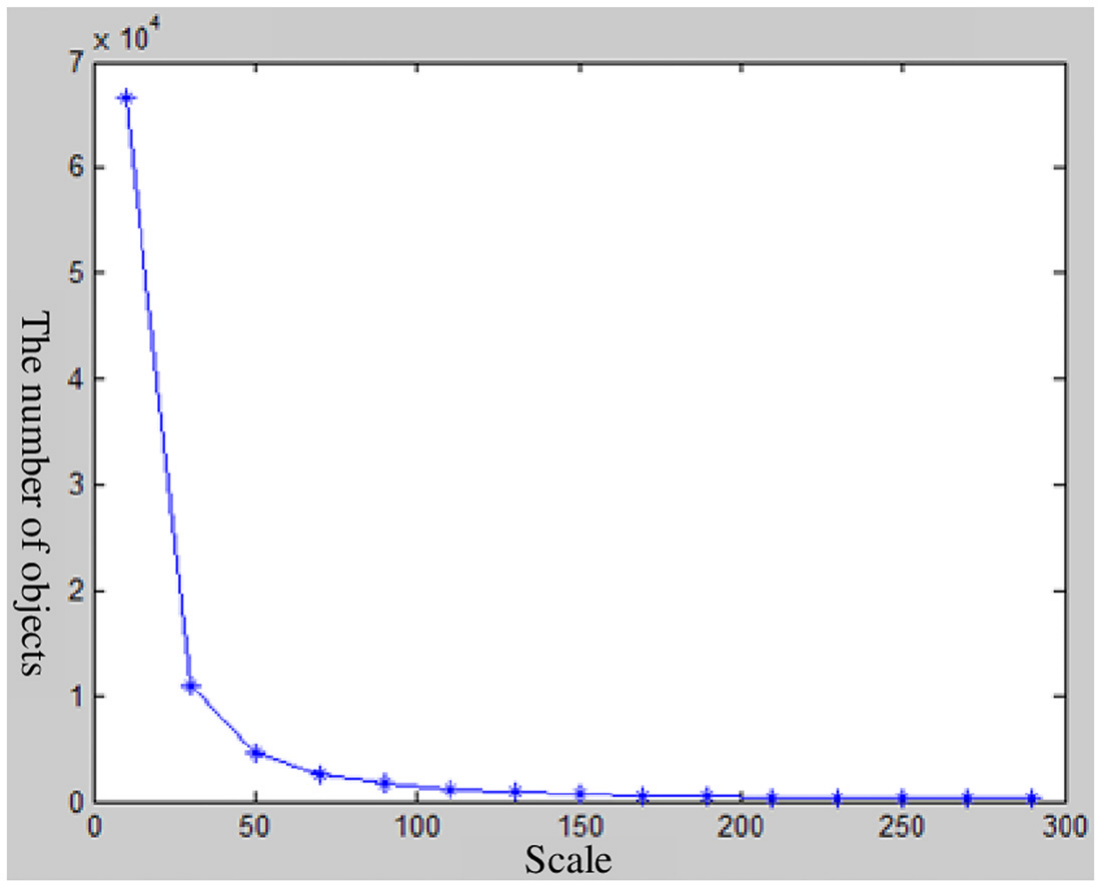
The relationship between the number ofobjects and segmentation scale.

**Fig 5:**
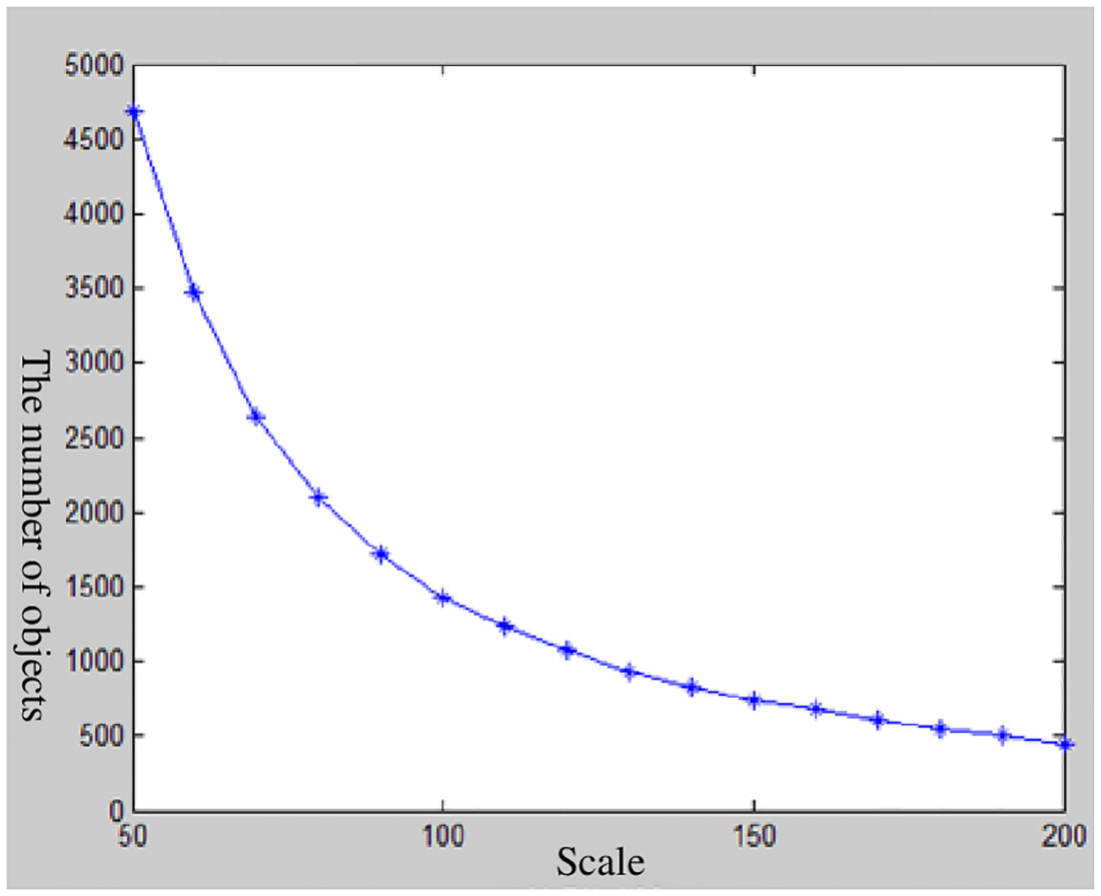
The selected of segmentation scale.

The image segmentation experiment is done by eCognition. The segmentation scale range is set to from 10 to 300, the step length is set to20, the shape factor is set to 0.3, and the tightness factor is set to 0.5. The initial segmentation sequence is then obtained. The segmentation sequence result is shown in Fig 4. The object segmentation number presents an exponential change when the segmentation dimensions are in the range of 10 to 50, whereas the object segmentation number presents little change when the segmentation scale is more than 200. Therefore, the segmentation scale range in this study is confirmed to be between 50 and 200, and the segmentation sequence result is shown in Fig 5.

3. Result analysis and discussion

**Fig 6:**
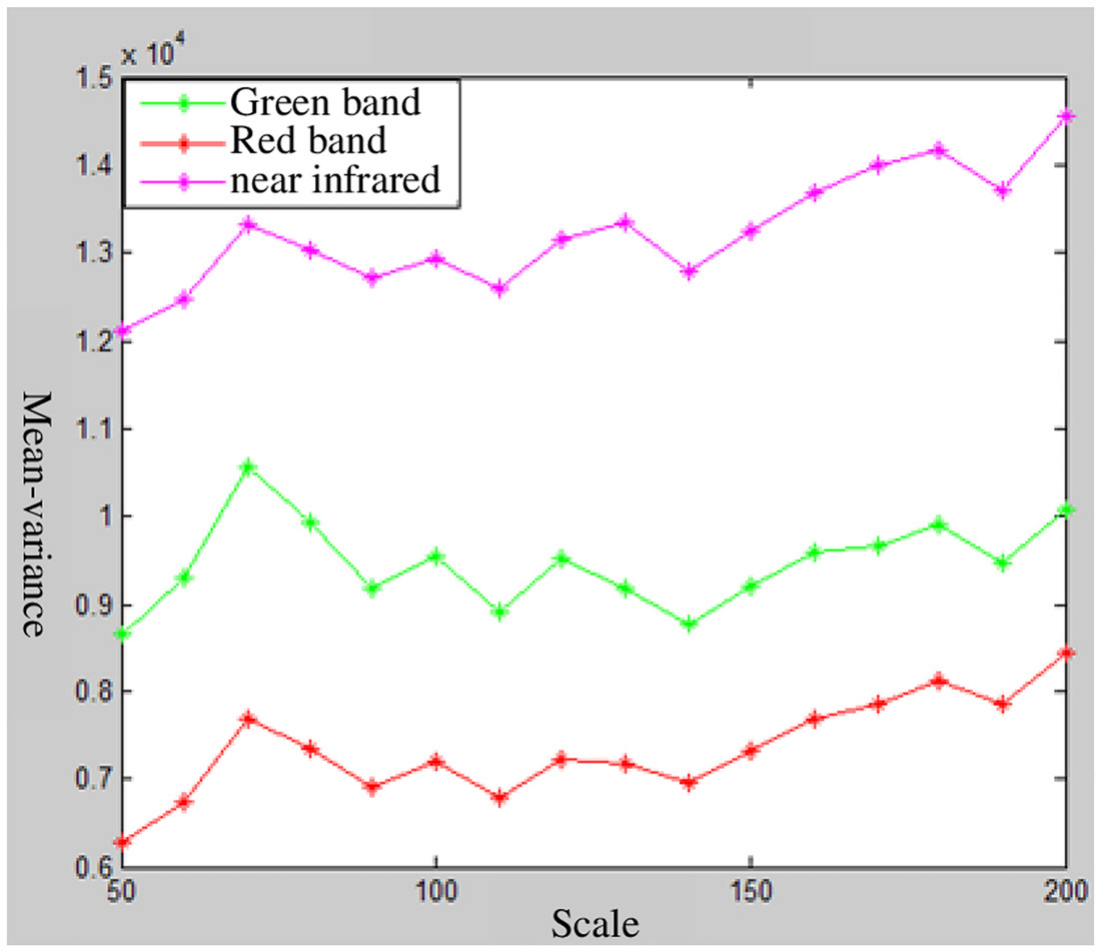
The relationship between mean-variance and segmentation scales for each band.

**Fig 7:**
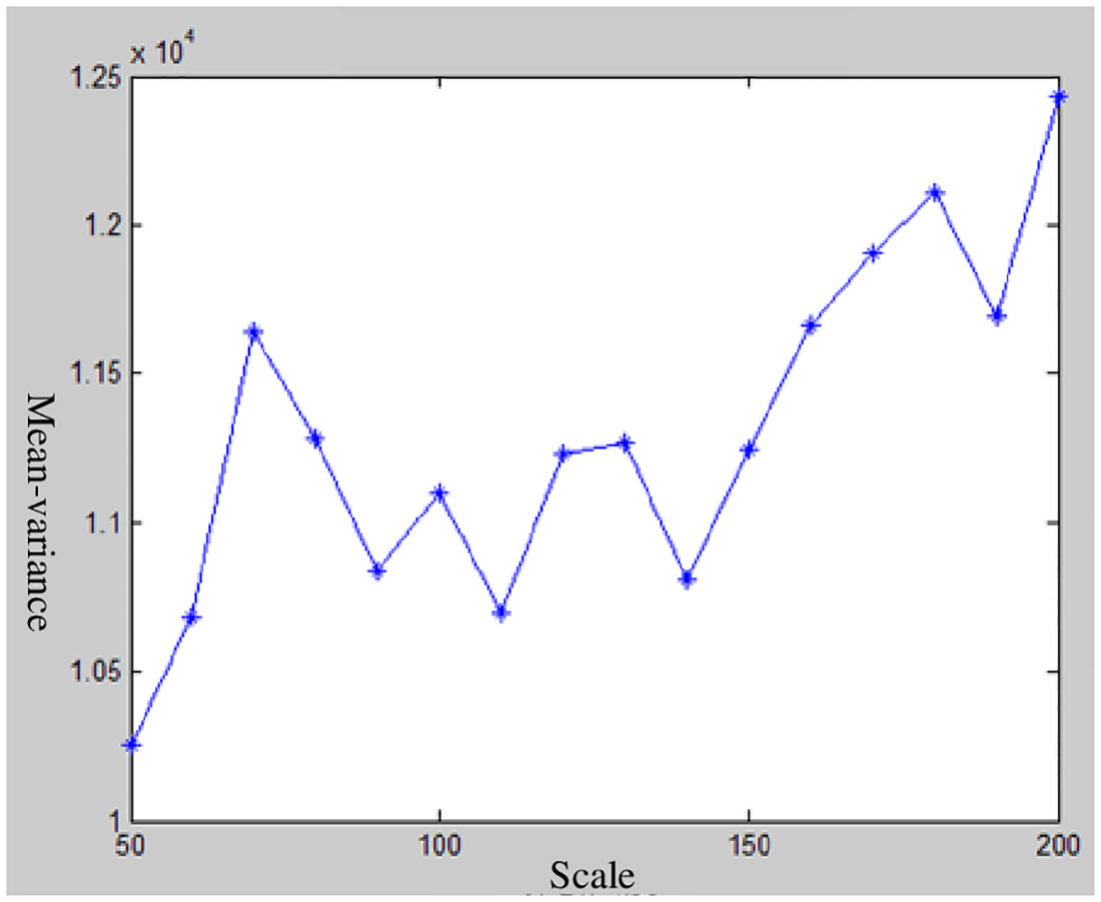
Relationship between the weighted mean-variance and segmentation scale.

With the use of the different scales, the attribute information of the polygon object in a different scale layer is obtained. Finally, the variance weighted average values and variance values of the objects in different image layers are calculated with the method above-mentioned. The corresponding curve graph between the variance or weighted variance value and the segmentation scale is drawn. The result is shown in Figs 6 and 7. The optimal segmentation scale values can be identified by analyzing the curve graph.

**Fig 8:**
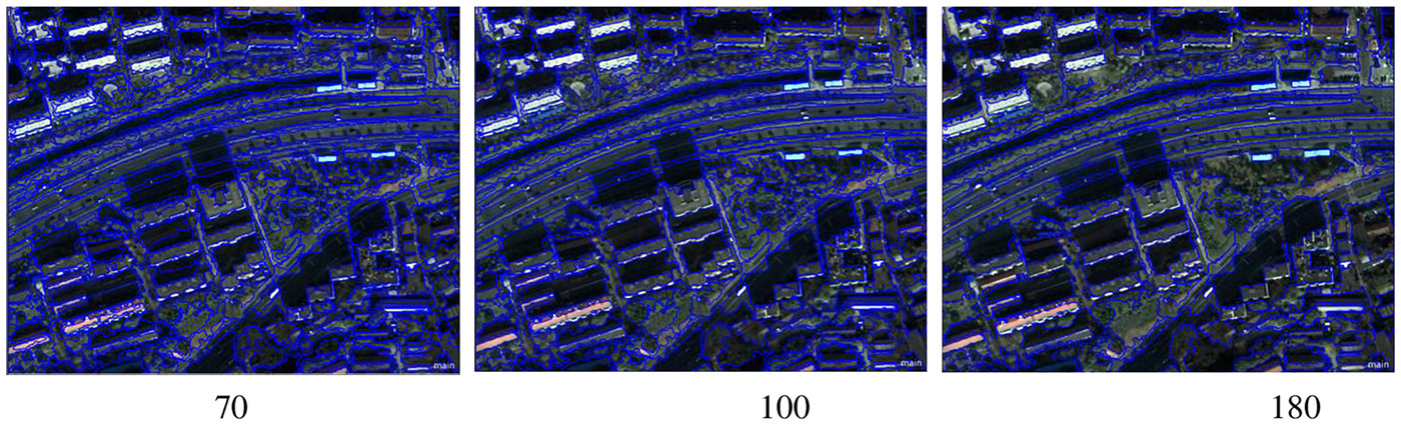
The result of different segmentation scales.

Figs 6 and 7 depict that the variance value of each band is large, but the curves are similar to one another. Many peaks also appear in the curve graph (Fig 7) and these peaks correspond to the segmentation scale of 70, 100, 120, 130 and 180. Fig 6 shows that the scale of 130 is a local peak in the near-infrared band, whereas the scale of 120 is a local peak in the green and red bands. These results illustrate that the scales of 120 and 130 are inconsonant among these three image bands. Thus, the segmentation scales of 70, 100, and 180 are selected for image segmentation. The image segmentation results that correspond to the three segmentation scales are shown in Fig 8.

From the analysis of Fig 8, we can conclude that the information on vegetation and bare patches in the segmentation result is comparatively complete with the use of the segmentation scale of 70. Most of the shadows and buildings in the segmentation results are extracted with the use of the segmentation scale of 100. The area and shape of the water object are not changed, whereas the roads are combined into large objects. Furthermore, their shapes become regular with the use of the segmentation scale of 180. Therefore, in the segmentation layer that uses as cale of 180, the information extraction of roads, water, and large areas of vegetation has an improved effect.

### Calculation of the shape and firmness factor

The quality of image segmentation is not only interrelated with the band weight and integral scale but also with the shape and firmness factors[15]. In the current study, the shape and firmness factors are calculated by the control variable method.

#### Methodology

The control variable method involves two steps:

1. The optimal segmentation scale value is set to the value corresponding to the feature, and its initial tightness factor value is set to 0.5 on the basis of the results of the optimal segmentation scale value in above-mentioned. The relation graph between the heterogeneity or homogeneity index value and the shape factor is drawn by a change in the shape factor parameters. The optimal parameters of the shape factor are obtained by analyzing the relation graph.
2. The basis of the optimal segmentation scale value, the optimal parameters of the shape factor, and the tightness factor parameters are changed. The relation graph between the heterogeneity or homogeneity index value and the tightness factor value is drawn. The optimal parameters of the tightness factor value are obtained by analyzing the relation graph.

#### Experiment and result analysis

1. Calculation of the shape factor.

The shape factor is set in the range of 0 to 0.8, and its step length is set to 0.1. The average segmentation evaluation index (ASEI) is used to determine the optimal parameters of the shape factor. The ASEI calculation formula is presented as Formulas (5) to (8):

$${\text{V}}_{\text{L}}=\frac{\sum_{\text{i}=1}^{\text{n}}{\text{a}}_{\text{i}}{\text{v}}_{\text{i}}}{\sum_{\text{i}=1}^{\text{n}}{\text{a}}_{\text{i}}}$$ (5)

$$\Delta {\text{C}}_{\text{L}}=\frac{1}{\text{l}}\Sigma_{i=1}^{n}\Sigma_{j=1}^{m}{\text{l}}_{\text{ij}}\left|\bar{{\text{C}}_{\text{L}}}-{\text{C}}_{\text{Li}}\right|$$ (6)

$${\text{ASEI}}_{\text{L}}=\frac{\Delta {\text{C}}_{\text{L}}}{{\text{V}}_{\text{L}}}$$ (7)

$${\text{ASEI}}_{\text{I}}=\Sigma_{L=1}^{m}{\text{w}}_{\text{L}}{*\text{ASEI}}_{\text{L}}$$ (8)

In Formula (5), v_i_ is the standard deviation of object, a_i_ is the area of object i, and n is the number of objects in the experimental region. In Formula (8), n is the number of objects in the experimental region, ΔC_L_ is the absolute value of the difference between the object and the neighborhood pixel mean value in the band L, C_Li_ is the pixel value of i in the band L, $\bar{{\text{C}}_{\text{L}}}$ is the pixel mean value of one object in the band L, m is the number of objects neighboring the target object, l is the boundary length of the target object, and l_ij_ is the boundary length of objects i and j. In Formulas (7) and (8), ASEI_L_ is the heterogeneity index of band L, and ASE_I_ is the heterogeneity index of all bands.

**Fig 9:**
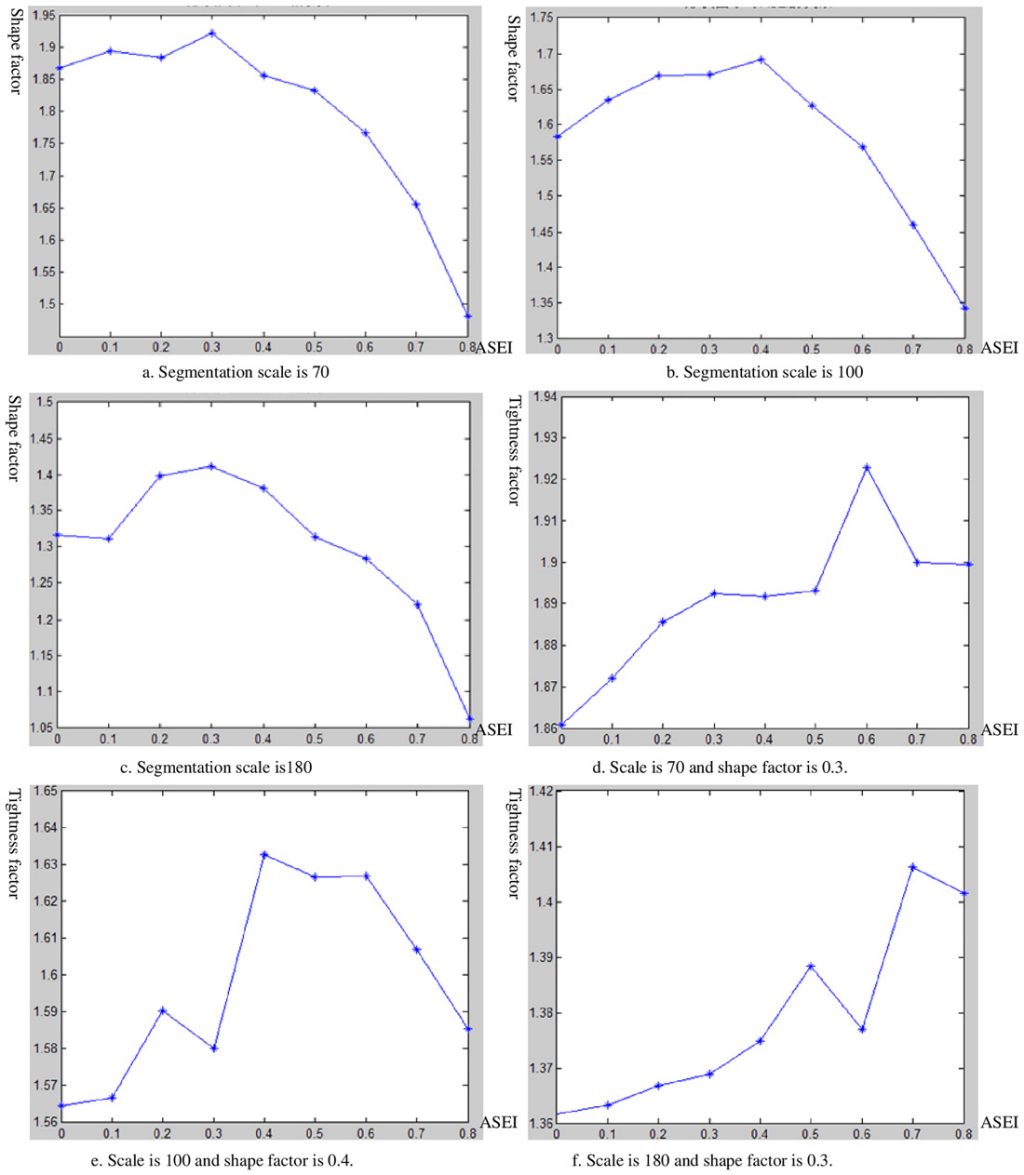
a-c: The relationships between shape factor of each segmentation scale and ASEI. d-f: The relationships between compactness factor of each segmentation scale and ASEI.

The ASEI value is calculated by the formulas from formulas (5) to (8), and the relation graph between the shape factor and ASEI, which is shown in (Fig 9a–9c), is drawn. In (Fig 9a–9c), these coordinate figures of the horizontal and vertical axis represent the shape factor and ASEI values. The analysis of (Fig 9a–9c) shows that the optimal shape factor values are 0.3, 0.4, and 0.3, which correspond to the optimal segmentation scale values, such as 70, 100, and 180.

3. Calculation of the tightness factor

The band weighting factor, optimal segmentation scale, and its corresponding optimal shape factor parameter are unchanged. The tightness factor is set in the range of 0 to 0.8, and the step length is set to 0.1. ASEI is used to determine the optimal parameters of the tightness factor. The ASEI calculation is shown as the formulas from formulas (5)) to (8). The relation graph between the tightness factor and ASEI is shown in (Fig 9d–9f). In (Fig 9d–9f), the coordinate figures of the horizontal and vertical axes represent the tightness factor and ASEI values. By analyzing (Fig 9d–9f), we can find that the directions of the curve are changed, and that multiple local peaks appear.

## Classification Result and Analysis

### Calculation results of the optimal segmentation parameters

According to the calculation method and the experimental process described as above-mentioned, the specific parameters of the optimal segmentation results are obtained. These results are listed in Table 5. The optimal correspondence of the three parameters is left three columns Table 5.

**Table 5:** The extraction accuracy corresponding to its optimal segmentation parameters.

scale	Shape factor	tightness factor	feature	extraction accuracy
70	0.3	0.6	Vegetation	84.55%
70	0.3	0.6	bare land	71.43%
100	0.4	0.4	shadow	86.67%
100	0.4	0.4	Buildings	86.79%
180	0.3	0.7	Roads	82.35%
180	0.3	0.7	water	82.35%

### Classification based on image optimal segmentation parameters

This study has obtained the features segmentation results by using the optimal segmentation parameters. The accuracy of each feature classification is right two columns Table 5.

In the process of multi-scale segmentation classification, image segmentation was realized with the use of a multi-level segmentation technology, and the network hierarchy of the image object was established on the basis of the segmentation results. Finally, the feature information was extracted by selecting the optimal segmentation on different object layer parameters and spectral characteristics. This research area is mainly divided into three parts. Given that the roads and water are spread out over a large area, and they present an aggregate distribution, the first layer contains water and road information and is represented by few objective polygons. The second layer is the sub-layer of the first layer, which is used to extract building and shadow information. The third layer is the sub-layer of layers 1 and 2, and is used to extract vegetation and bare land information. A few separate polygons are used to represent this feature information.

### Classification result analysis and discussion

**Fig 10:**
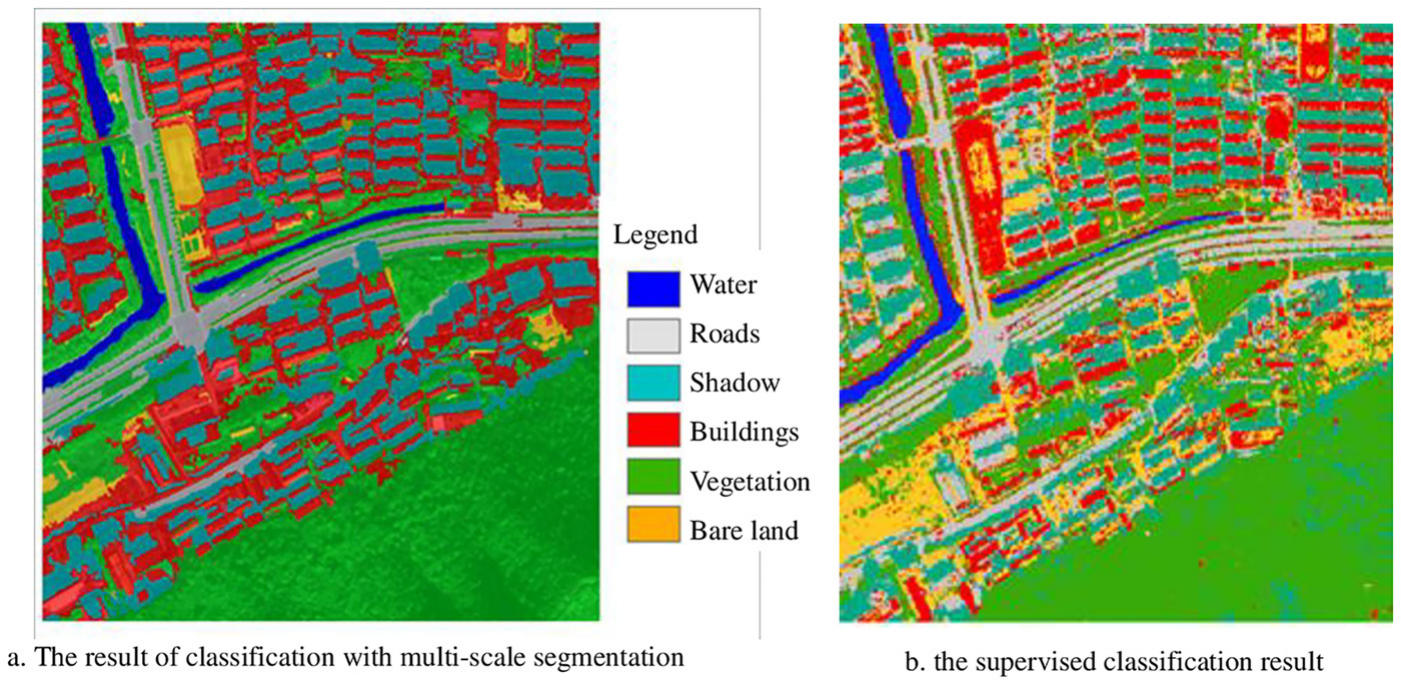
Two kinds of classification results.

The multi-scale segmentation classification result of the experiment data are shown in Fig 10a. To verify that the classification accuracy is improved, the supervised classification result of the experiment data is calculated Fig 10b.

To verify the reliability of the multi-scale segmentation classification result, the confusion matrixes of the classification results for the feature misclassification rates are calculated, and the results are shown in Table 6. The data in Table 6 show that the misclassification rates of all features of the supervised classification result, particularly buildings and vegetation, are higher than those of the multi-scale segmentation classification result. Thereafter, the degree of accuracy of the two classification result is calculated. The overall accuracy of the multi-scale segmentation classification result is 89.55%, and its Kappa coefficient is 0.862. While the overall accuracy of supervised classification result is 70.45% and its Kappa coefficient is 0.626. The accuracy of the multi-scale segmentation classification result is considerably improved compared with the supervised classification result. The comparison result indicates that the multi-scale segmentation classification and the optimal extraction parameters are effective. The multi-scale segmentation classification method can maximize the use of the spectrum, the structure feature, and texture information. For example, roads and buildings use the structure characteristics of the aspect ratio; thus the accuracy of the information extraction is considerably improved.

**Table 6:** The confusion matrix of multi-scale segmentation and supervised classification result.

Feature class	Vegetation		Water		Roads		Shadow		Buildings		Bare land	
	O	S	O	S	O	S	O	S	O	S	O	S
Vegetation	**97**	**82**	0	0	3	3	2	5	5	3	0	0
water	0	0	**12**	**11**	0	0	0	0	0	0	0	0
Roads	0	4	0	0	**29**	**28**	0	1	0	20	0	0
shadow	0	12	5	3	0	0	**56**	**49**	6	1	0	0
Buildings	4	2	0	3	4	5	1	4	**94**	**55**	2	3
bare land	1	2	0	0	0	0	0	0	1	27	**12**	**11**

Note: in Table 6, the letter O indicates multi-scale segmentation classification result, and the letter S indicates supervised classification result.

## Discussion and Conclusion

In this study, the optimal segmentation parameters method of object-oriented image segmentation and high-resolution image information extraction are examined in depth. For the WorldView-2 experimental data, the Panimage sharpening fusion method is the optimal method. It does not only maintain the original image spectral information but also enhances the image texture and spatial information. OIF indicates bands 7, 5, and 3 are the best combination of object-oriented image segmentation, and that their segmentation weights are 3, 1, and 1, respectively. The improved weighted variance method is proposed to calculate the optimal segmentation scale, and this scale is selected with the use of such a method. The optimum shape factor parameters and the tightness factor are calculated by the application of the control variable method and by using the numerical results of the heterogeneity and homogeneity indexes. The experiment results indicate that the vegetation and bare land extraction effect are the best when the segmentation scale is set to 70, the shape parameter is set to 0.3, and the tightness factor is set to 0.6. Buildings and the shadow extraction effect show the best results when the segmentation scale is set to100, the shape parameter is set to 0.4, and the tightness factor is set to 0.4. Finally, roads and the water extraction effect are the best when the segmentation scale is set to180, the shape parameter is set to 0.3, and the tightness factor is set to 0.7. Multi-scale segmentation is realized for the image according to the calculated optimal parameters, and the level of hierarchical network structure is established. An 89.55% overall accuracy of extraction and 0.862 Kappa coefficient are achieved when the rules of information extraction are set, and object-oriented information extraction is conducted. As a result, information extraction is significantly improved. These results indicate that the proposed information extraction of object-oriented high-resolution remote sensing images based on the optimal segmentation parameters is highly effective and has practical value.

We propose an objective method that uses the inherent properties of remote sensing data (object autocorrelation and variance) to support the calculation of segmentation parameters. The proposed method allows users to benefit from the potential of object-based classification methods for extracting information from high-resolution satellite images. Therefore, further researches are expected to realize the automaticity of the method, particularly the use of the remote sensing software tools such as eCognition. The optimal segmentation parameter calculation is based on the scale parameter sequence, and it has some limitations. So, we still need to further study the dimension parameters on image segmentation results of comprehensive evaluation index, the optimal segmentation scale and scope in the future. In addition, the relationship between the image resolution and the optimal parameters, especially the scale parameter, should be further examined in the future work.

## Supporting Information

S1 Dataset This is the raw data of image enhancement processing results, and it also is the raw data of fig 2. (ZIP)

S2 Dataset This is the raw data of the subset dataresults, and it also is the raw data of fig 3. (ZIP)

S3 Dataset The raw data of segmentation and classification experiments. (ZIP)

S4 Dataset The figures of this article. (ZIP)
