# Supplementary figures and images for: Information Extraction of High Resolution Remote Sensing Images Based on the Calculation of Optimal Segmentation Parameters
Source: PLoS One. 2016 Jun 30;11(6):e0158585. doi: 10.1371/journal.pone.0158585 (PMC4928919; doi:10.1371/journal.pone.0158585)

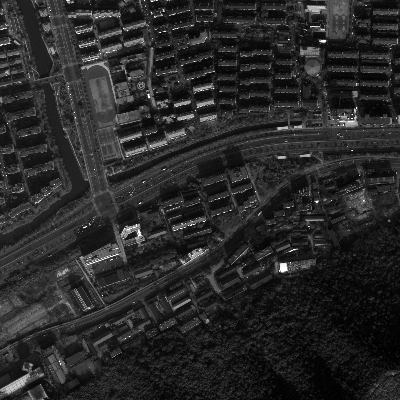

Supplement: S2 Dataset — (ZIP) [file pone.0158585.s002.zip › Fig.3/pan-cut.tif.ovr]

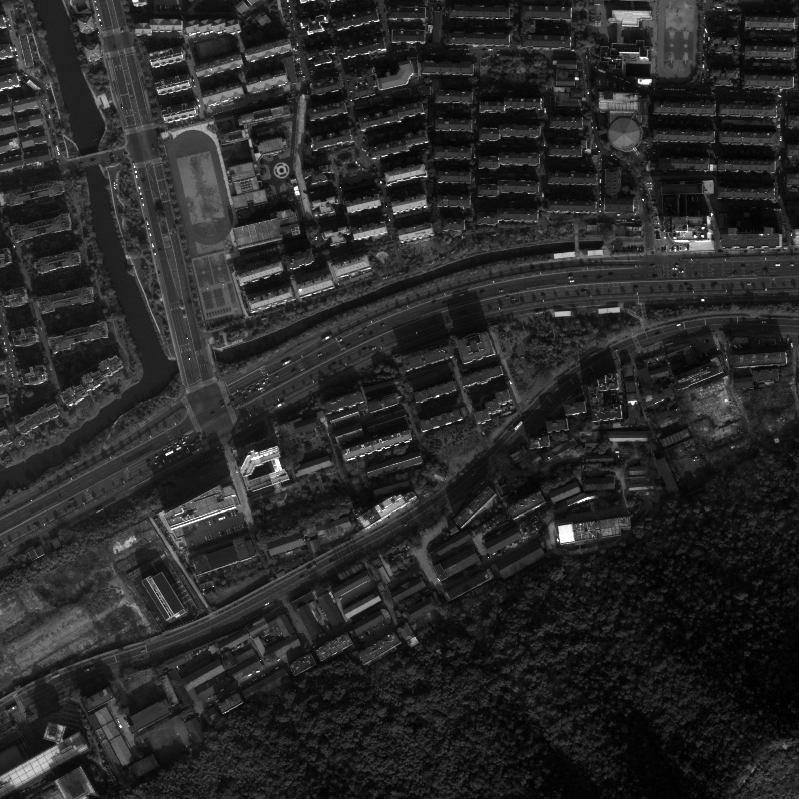

Supplement: S2 Dataset — (ZIP) [file pone.0158585.s002.zip › Fig.3/pan-cut.tif.tif.ovr]

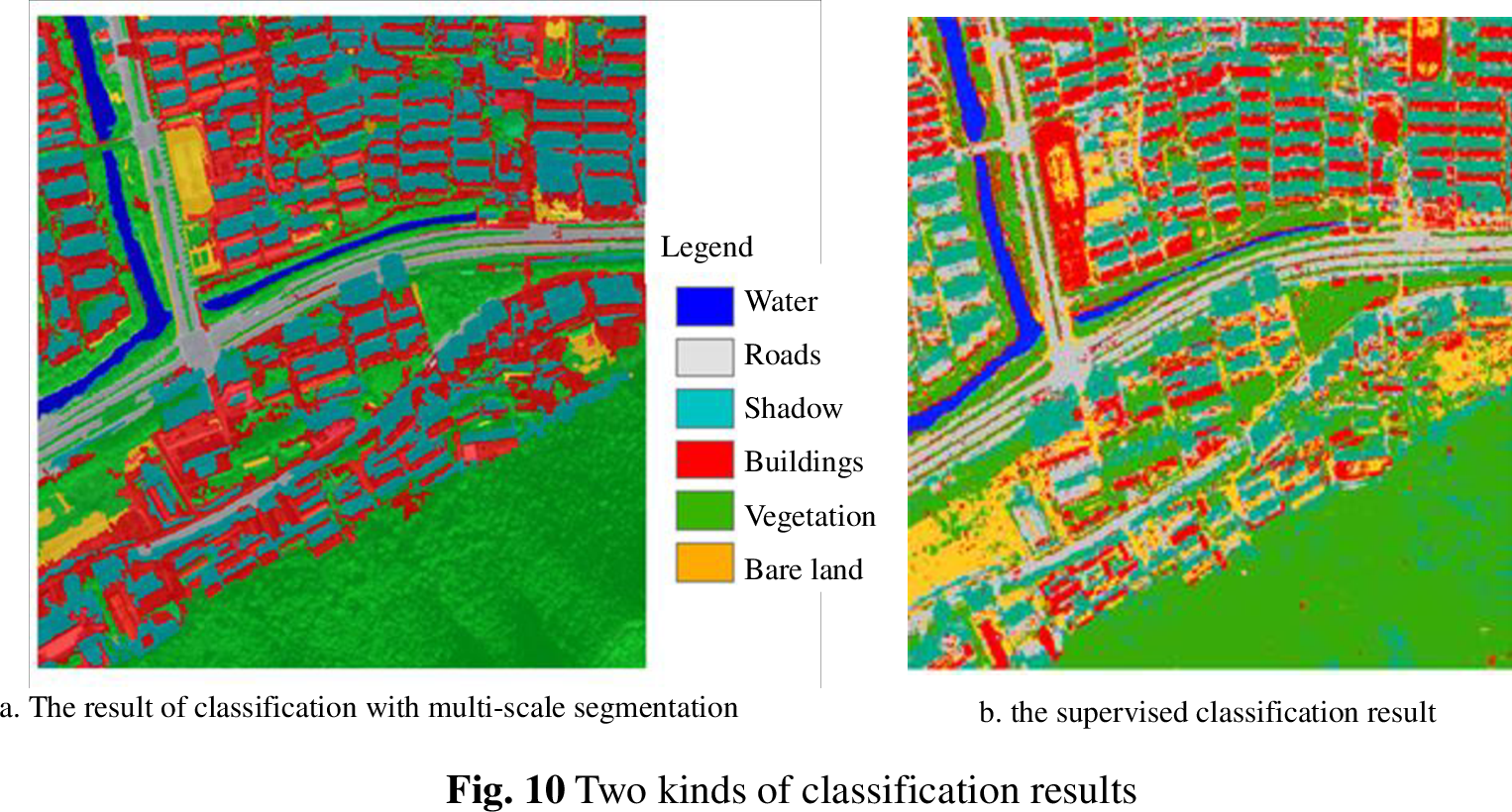

Supplement: S4 Dataset — (ZIP) [file pone.0158585.s004.zip › figure10.tif]

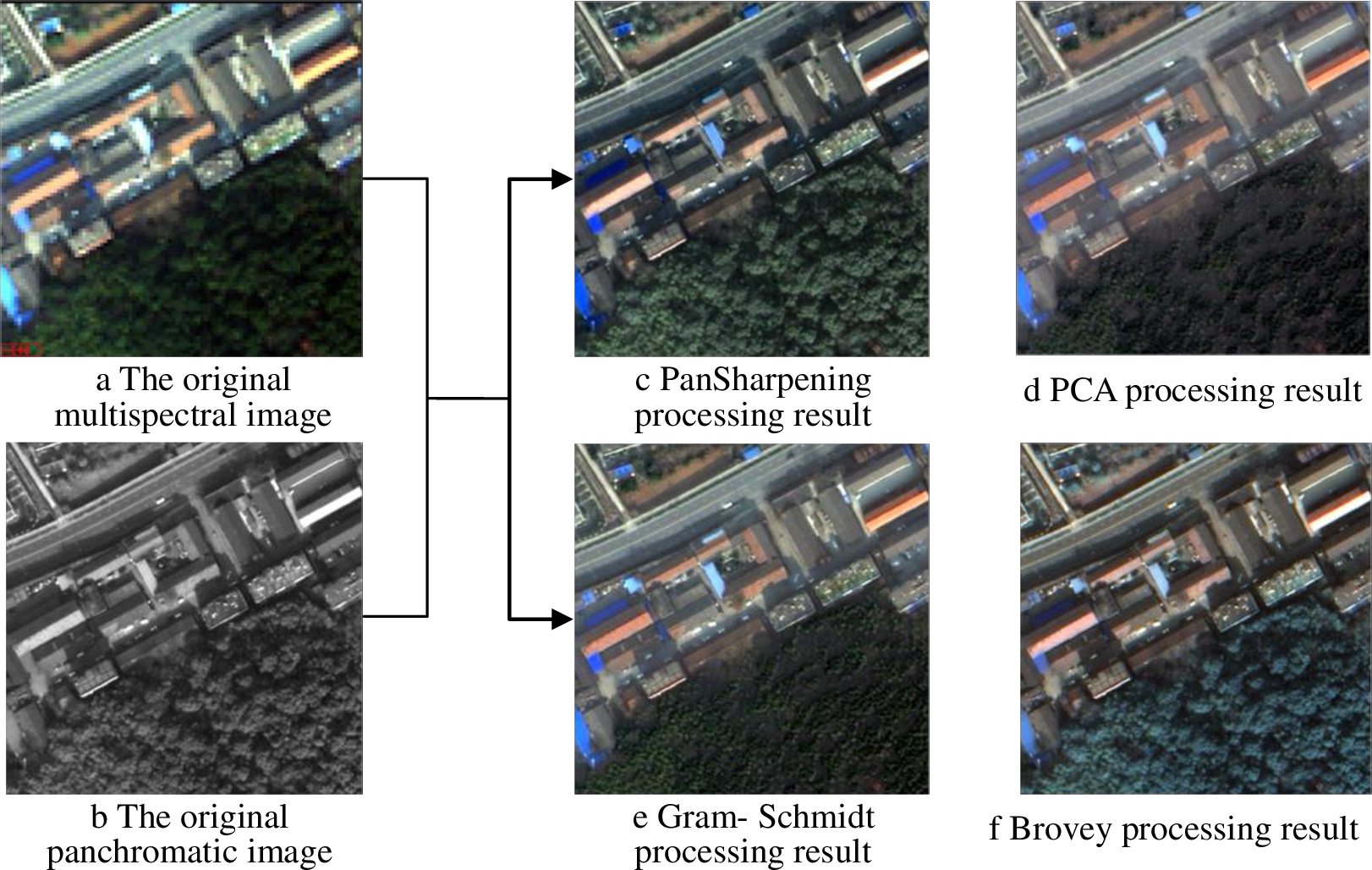

Supplement: S4 Dataset — (ZIP) [file pone.0158585.s004.zip › figure2.tif]

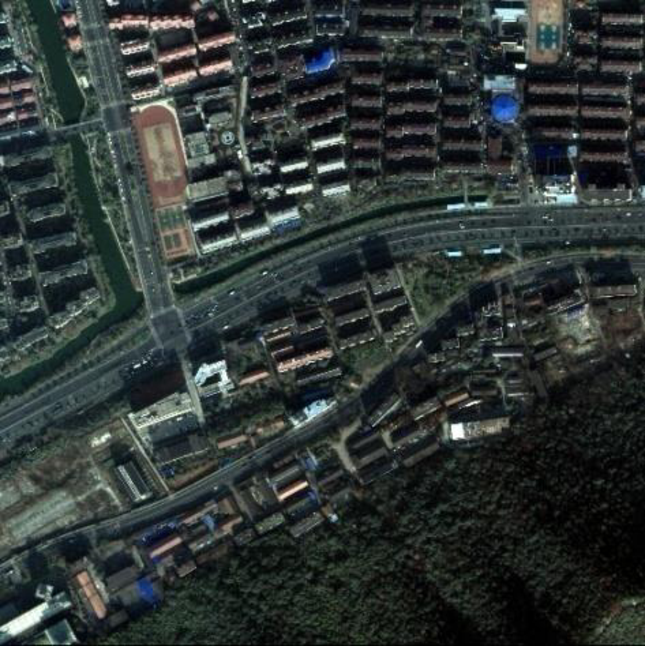

Supplement: S4 Dataset — (ZIP) [file pone.0158585.s004.zip › figure3.tif]

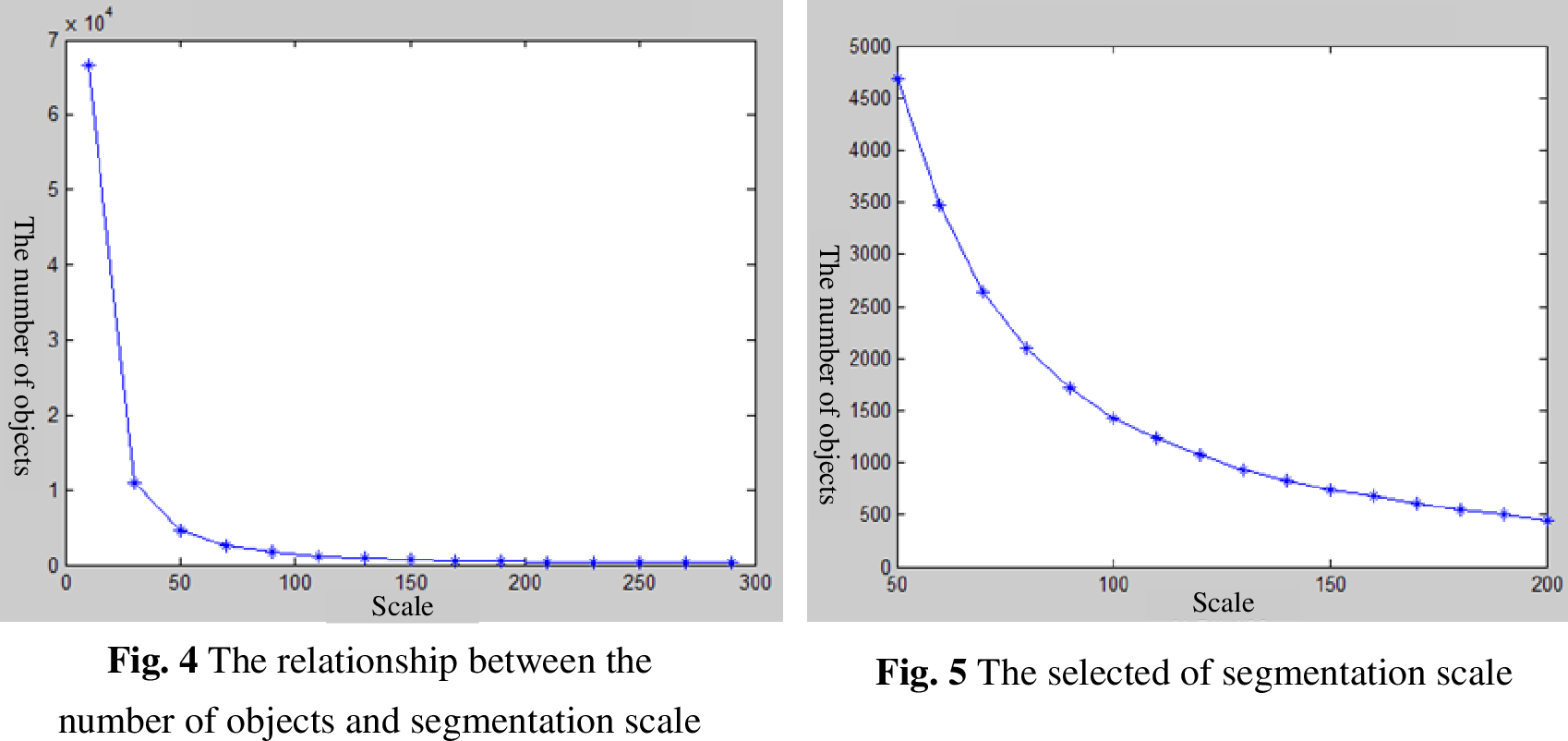

Supplement: S4 Dataset — (ZIP) [file pone.0158585.s004.zip › figure4-5.tif]

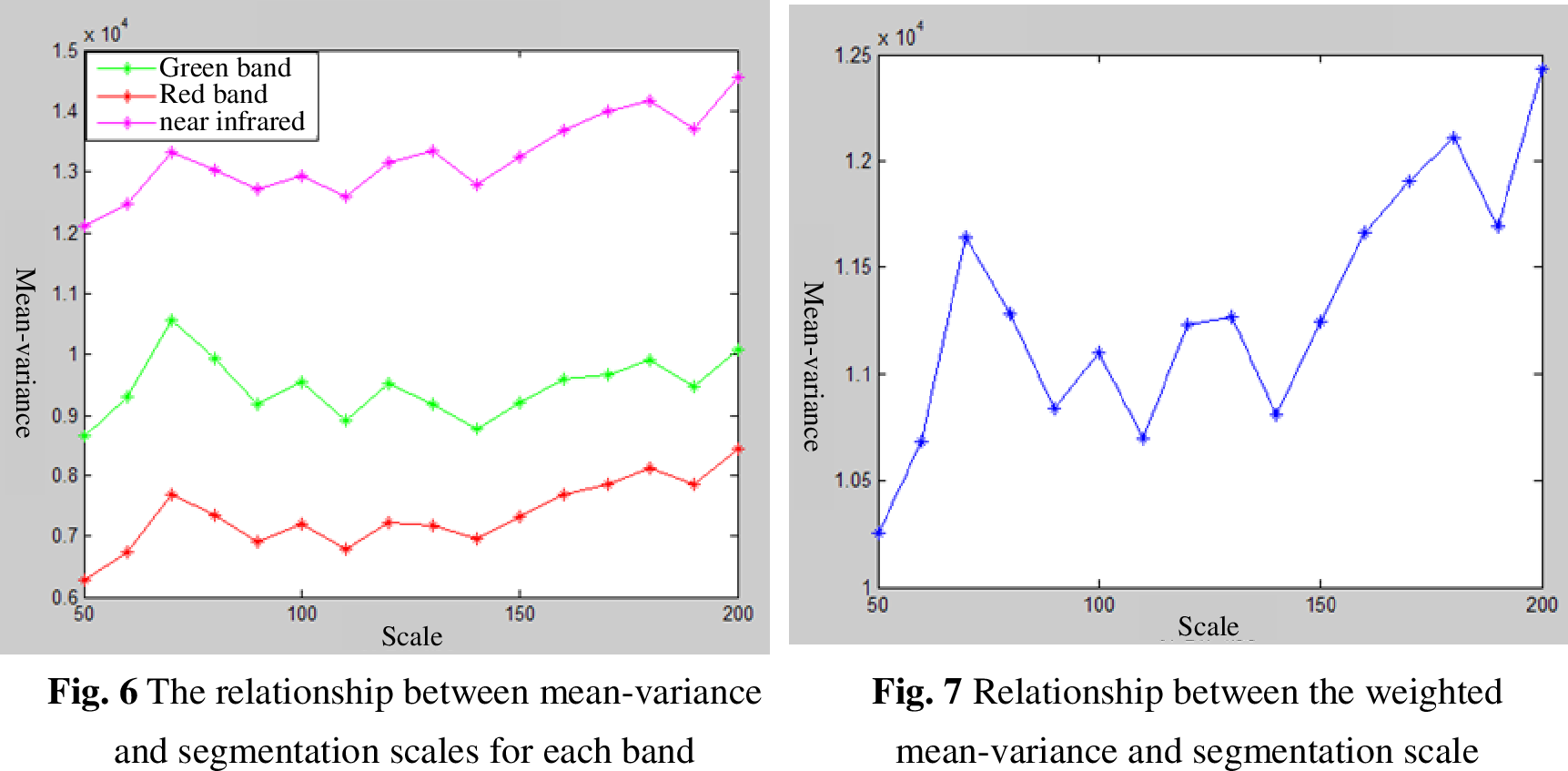

Supplement: S4 Dataset — (ZIP) [file pone.0158585.s004.zip › figure6-7.tif]

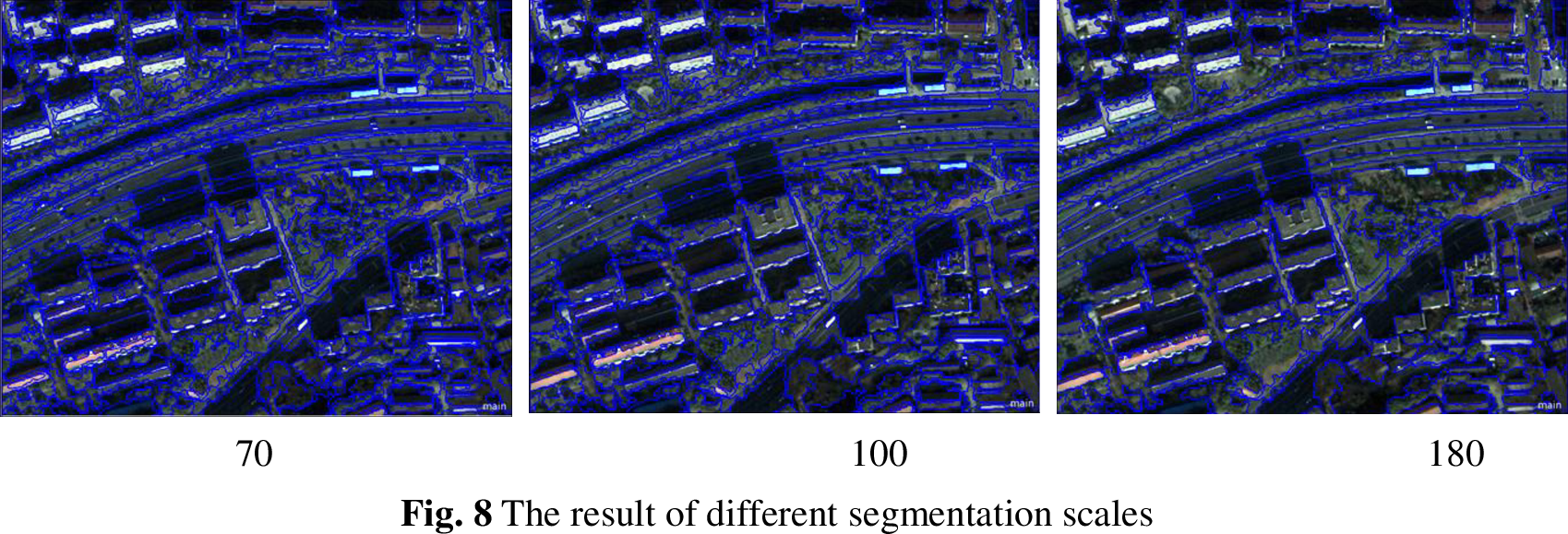

Supplement: S4 Dataset — (ZIP) [file pone.0158585.s004.zip › figure8.tif]

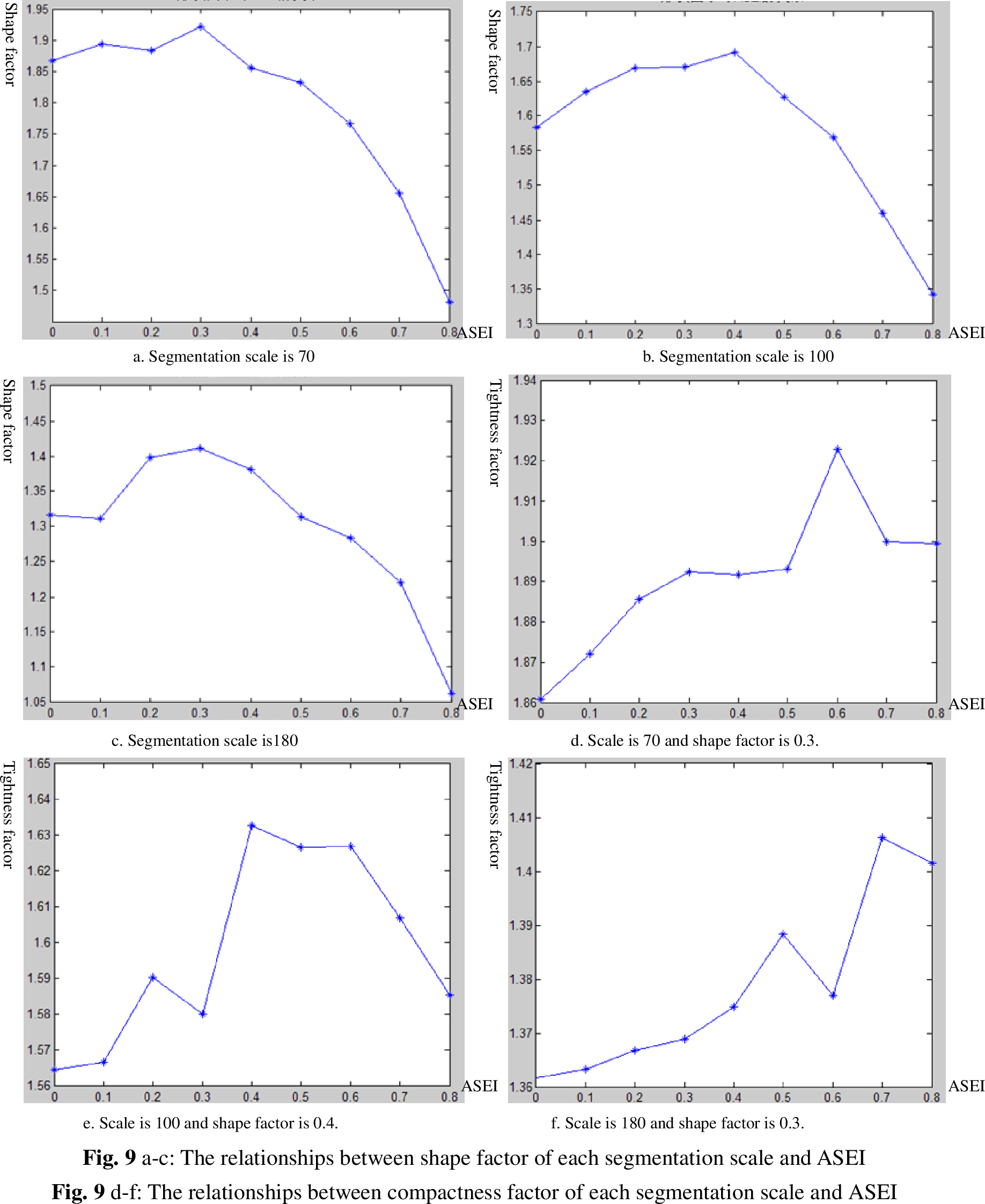

Supplement: S4 Dataset — (ZIP) [file pone.0158585.s004.zip › figure9.tif]

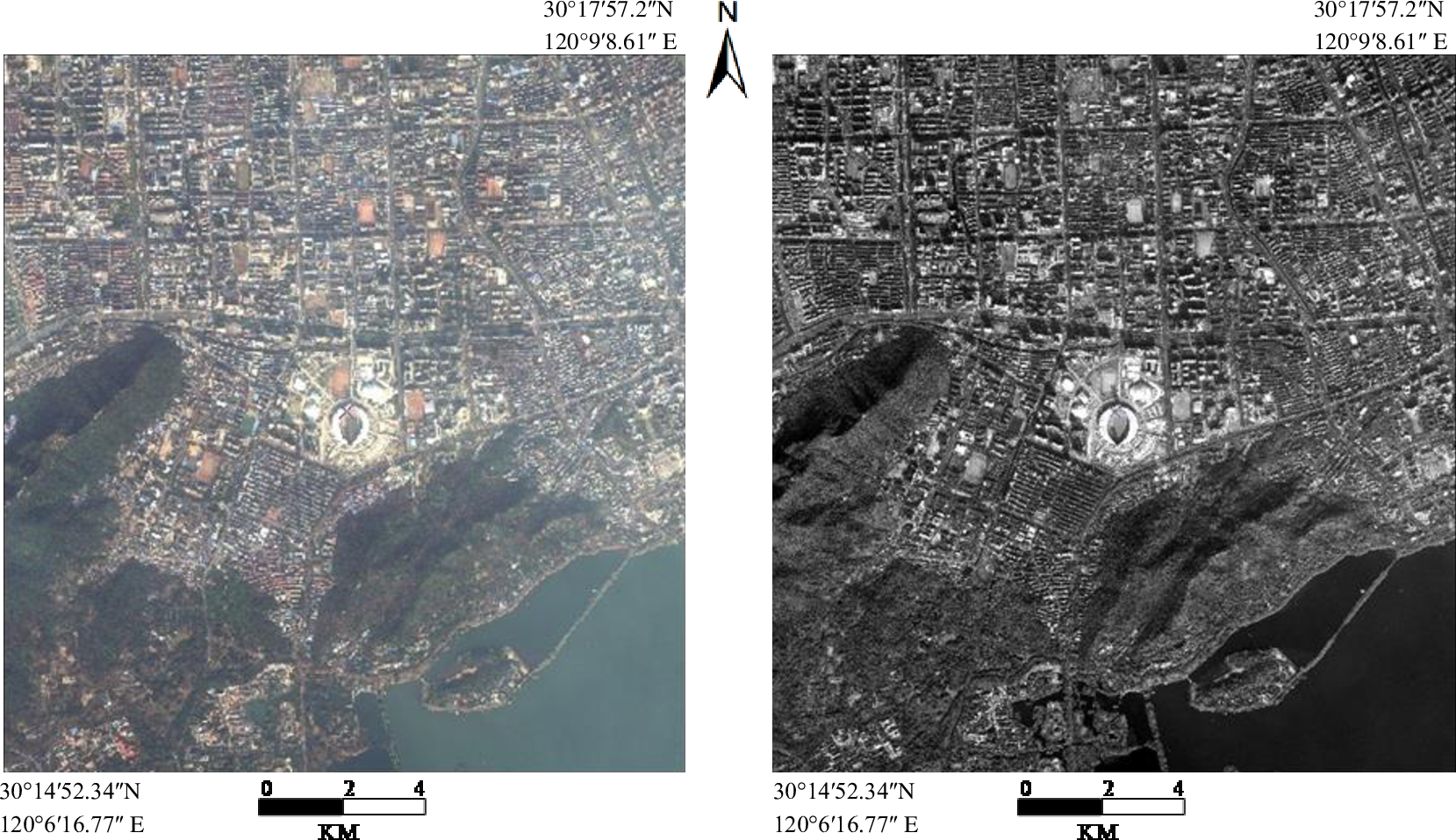

Supplement: S4 Dataset — (ZIP) [file pone.0158585.s004.zip › figure1.tif]
